# A Health Information Quality Assessment Tool for Korean Online Newspaper Articles: Development Study

**DOI:** 10.2196/24436

**Published:** 2021-07-29

**Authors:** Naae Lee, Seung-Won Oh, Belong Cho, Seung-Kwon Myung, Seung-Sik Hwang, Goo Hyeon Yoon

**Affiliations:** 1 Department of Public Health Science Graduate School of Public Health Seoul National University Seoul Republic of Korea; 2 Department of Family Medicine Healthcare System Gangnam Center Seoul National University Hospital Seoul Republic of Korea; 3 Department of Family Medicine Seoul National University College of Medicine Seoul Republic of Korea; 4 Department of Family Medicine Seoul National University Hospital Seoul Republic of Korea; 5 Department of Cancer Biomedical Science National Cancer Center Graduate School of Cancer Science and Policy Goyang Republic of Korea; 6 Division of Cancer Epidemiology and Management Research Institute, National Cancer Center Goyang Republic of Korea; 7 Department of Family Medicine and Center for Cancer Prevention and Detection Hospital, National Cancer Center Goyang Republic of Korea; 8 Liver Korea Seoul Republic of Korea

**Keywords:** assessment tools, information seeking, newspaper articles, online health information, quality assessment

## Abstract

**Background:**

Concern regarding the reliability and accuracy of the health-related information provided by online newspaper articles has increased. Numerous criteria and items have been proposed and published regarding the quality assessment of online information, but there is no standard quality assessment tool available for online newspapers.

**Objective:**

This study aimed to develop the Health Information Quality Assessment Tool (HIQUAL) for online newspaper articles.

**Methods:**

We reviewed previous health information quality assessment tools and related studies and accordingly developed and customized new criteria. The interrater agreement for the new assessment tool was assessed for 3 newspaper articles on different subjects (colorectal cancer, obesity genetic testing, and hypertension diagnostic criteria) using the Fleiss κ and Gwet agreement coefficient. To compare the quality scores generated by each pair of tools, convergent validity was measured using the Kendall τ ranked correlation.

**Results:**

Overall, the HIQUAL for newspaper articles comprised 10 items across 5 domains: reliability, usefulness, understandability, sufficiency, and transparency. The interrater agreement for the article on colorectal cancer was in the moderate to substantial range (Fleiss κ=0.48, SE 0.11; Gwet agreement coefficient=0.74, SE 0.13), while for the article introducing obesity genetic testing it was in the substantial range, with values of 0.63 (SE 0.28) and 0.86 (SE 0.10) for the two measures, respectively. There was relatively low agreement for the article on hypertension diagnostic criteria at 0.20 (SE 0.10) and 0.75 (SE 0.13), respectively. Validity of the correlation assessed with the Kendall τ showed good correlation between tools (HIQUAL vs DISCERN=0.72, HIQUAL vs QUEST [Quality Evaluation Scoring Tool]=0.69).

**Conclusions:**

We developed a new assessment tool to evaluate the quality of health information in online newspaper articles, to help consumers discern accurate sources of health information. The HIQUAL can help increase the accuracy and quality of online health information in Korea.

## Introduction

With advancements in technology, public interest toward health has increased. This has led to the public actively seeking health-related information and enhancing their medical expertise beyond simply managing their diseases [1], which has had a positive impact on health-related behaviors and beliefs [2]. Unfortunately, validating the accuracy of information can be difficult because there is enormous asymmetry of health-related information among providers and consumers [3]. The asymmetry of this information further creates a gap between consumers, expressed through the consumers’ health literacy or the production and distribution of inaccurate information [4,5]. To mediate this gap, the assessment of the quality of health-related information and the subsequent provision of the results to both providers and consumers must be undertaken using a standardized assessment tool. This will allow consumers to identify reliable information and reduce the risk of distributing channels of inaccurate health-related information [6,7].

There are various tools used to assess the quality of health-related information, including the DISCERN instrument, created by the University of Oxford [8]; the Health on the Net Foundation Code of Conduct (HONcode), developed by the Health on the Net Foundation in Switzerland [9]; and MedCERTAIN, supported by the Action Plan for Safer Use of the Internet of the European Union [10]. In the Republic of Korea, several tools to assess the quality of health-related information on the internet have also been developed [11-13]. Despite this, some of these instruments have not been designed to evaluate the quality of the information. Most of the tools do not evaluate online health information from newspaper articles, and their validity and reliability have not been verified [7,9,11,13]. Furthermore, these tools have a specific targeted format, thereby making it difficult to apply them to other media [12]. There are no gold-standard quality assessment tools for online health information [14]. DISCERN is a proven tool for validity and interrater reliability, but the validity and reliability of the Korean version has not been confirmed. Additionally, it is limited in the scope of application, as it is focused only on treatment information and is not applicable to online content about other aspects of health and illness, such as prevention and diagnosis, commonly covered in newspapers [15]. QUEST (Quality Evaluation Scoring Tool), which was recently developed for evaluating online health information, has also been proven to be valid, but it has not yet been translated into Korean [6], which makes it difficult to evaluate online health information in Korea.

With the increased use of the internet for information dissemination, the numbers of online newspaper articles and users have rapidly increased. According to a survey conducted in 2018, 63% of 3425 participants indicated that they preferred using the web to receive news, while only 17% of them chose print newspapers [16]. The results were particularly pronounced among younger generations. In 2016, the Nielsen Scarborough study noted that 49% of people accessed the internet to read newspaper articles in digital form instead of print [17].

According to the Korea Press Foundation, this trend can be seen in Korea as well, where 80.8% of the total population read newspaper articles via a mobile device [18]. As the number of online newspaper articles featuring health-related content has increased [19], there has been a rise in the number of problems stemming from inaccurate articles. This has created a need for addressing the quality assessment and management of health-related information [20]. A study that analyzed press reports on depression found that one-third of the articles did not mention the causes of depression at all, and only about half of the articles mentioned treatment methods [21]. Another study analyzed newspaper articles about sterility and found that most of them described infertile couples as abnormal or incomplete, consequently strengthening social prejudices [22]. Recently, it was reported that inaccurate newspaper articles can cause confusion among consumers when they are disseminated via social media [23]. Accordingly, the Association of Health Care Journalists suggested some fundamental principles to be followed when writing health-related articles, including professionalism, content, accuracy, independence, integrity, and responsibility [17]. Additionally, HealthNewsReview.org [24], a website that evaluates the quality of medical-related newspaper articles, has failed to describe in detail the process it adopts for developing criteria, and it has shown no evidence for individual criteria. Although the problems caused by health-related articles have increased, there are no suitable quality assessment tools to evaluate the quality of health-related newspaper articles in Korea. Therefore, this study aimed to develop the Health Information Quality Assessment Tool (HIQUAL) to assess health information in online newspaper articles.

## Methods

### Overview

**Figure 1:**
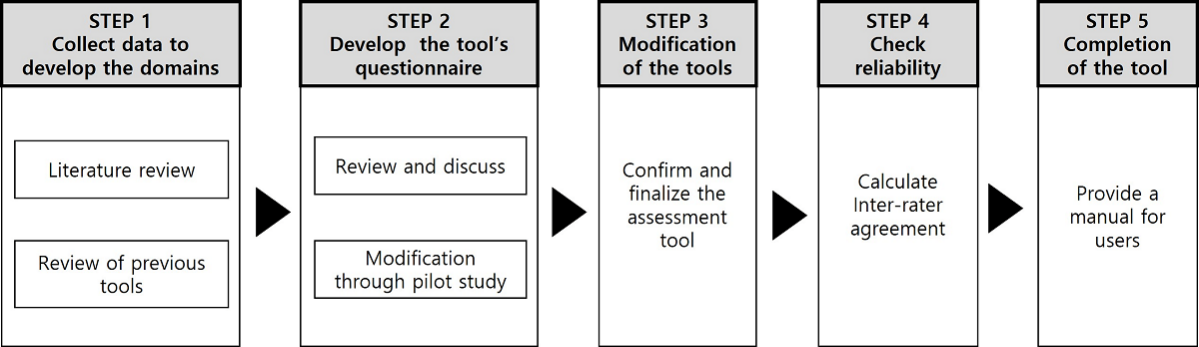
The process of developing the tool.

This study can be divided into four steps (Figure 1). First, we reviewed previous literature on the evaluation of health information quality assessment tools to develop the evaluation indicators. Second, we developed a draft of domains and questions through meetings and preliminary evaluations. Third, the assessment tool was modified and confirmed through evaluations and reviews at two different points in time. Fourth, we concluded the final agreement and validity with the assessment tool. The tool developed in this study—HIQUAL—was funded by the Korean Medical Association research project, which aims to develop standardized assessment tools and methods for systematic evaluation of health information from newspaper articles, television, and books.

### Review of Previous Health Information Quality Assessment Tools

A review of existing literature was conducted to select the domains that correspond to the content of the questions guiding the development of the HIQUAL. A study by Wang and Strong was used to classify various dimensions included in existing assessment tools [25]. In this study, the data quality (DQ) dimension was divided into several domains—*intrinsic*, *contextual, representational,* and *accessibility*—and presented in a hierarchical framework to understand DQ from a consumer's perspective. Intrinsic DQ means that facts have quality in their own right; contextual DQ emphasizes the situations that must be considered to assess the context of the information; representational DQ emphasizes a format that is concise and consistent and data whose meaning is understandable and interpretable; and accessibility DQ emphasizes the significance of the parts of the framework. Our study also used this category to organize domains of various previously developed evaluation tools.

Developed by Oxford University, the DISCERN instrument evaluates information on disease and treatment under 3 domains, including 8 reliability items, 7 quality of information items, and 1 comprehensive evaluation, and has established feasibility and reliability [8,25,26]. The HONcode, developed by the Health on the Net Foundation, offers 8 ethical codes that health information websites must follow: authority, complementarity, confidentiality, attribution, justifiability, transparency, financial disclosure, and advertising [9]. The American Medical Association provides guidelines for health information websites using 4 categories: content, advertising and sponsorship in online posting, privacy and confidentiality of site visitors, and effectiveness and security of e-commerce [27]. MedCERTAIN is a third-party certification system developed as part of a project supported by the European Union's Action Plan for Safer Use of the Internet. The assessment items consist of identification, feedback, operation, and site identification of “information providers,” as well as content, disclosure, policy, service, accessibility, and quality [10].

In Korea, Lee et al classified the common domains presented in previous studies as representation, contents, usage, and connection [28]. A study by Son referred to prior research and presented the evaluation criteria for health-related information websites in 7 domains: quality of content, authorship, purpose, design and aesthetic, functionality, contact address and feedback mechanism, and privacy [11]. The Internet Health Information Quality Checklist developed by Kim et al is characterized by different questions, depending on the user [12]. For professional use, it presents specific questions for items such as validity, sufficiency, and harmfulness of content, and the management is divided into purpose, authority, clarity of sponsorship, limitations of timing, commerciality, responsibility, privacy and security, ethics, and compliance. For general use, the question categories are divided into ease of understanding, adequacy, usefulness, sufficiency, appositeness, timeliness, admonition, ease of use, informativeness, and amusement [12]. The Korean Academy of Medical Sciences selected 5 evaluation criteria—reliability, usefulness, understandability, completeness, and publicity—and uses them for health information review certification projects [13]. The Health Information Monitoring Project of the Korean Medical Association uses a set of 6 criteria: scientific soundness, usefulness, sufficiency of information, whether facts are exaggerated, ease of use, and advertising. The Korea Institute for Health and Social Affairs conducted an evaluation of internet health information based on a set of 8 criteria: purpose (obviousness), appropriateness, accuracy, reliability, ease of use, authority, communication, and persistence [29].

### Development of the Draft of the HIQUAL

**Figure 2:**
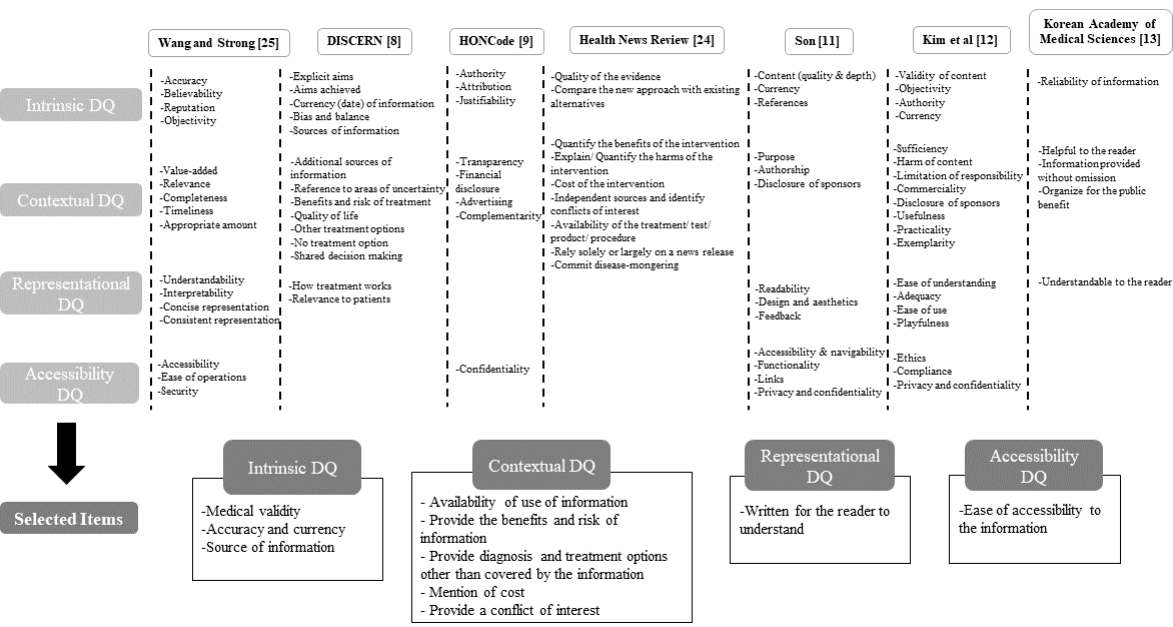
Selection of the domain through comparison of existing evaluation tools. DQ: data quality; HONCode: Health on the Net Foundation Code of Conduct.

The dimensions of the existing tools were classified according to the categories used by Wang and Strong (Figure 2) [25]. The indicators were assessed for the importance given to quality in online newspapers, providing the basis for the indicators and domains to be included in the new evaluation tool. Since the subject of the assessment tool was newspaper articles that are available in portal websites, the domain corresponding to accessibility was excluded, while draft questions were created for the other 3 domains. A rater with expertise in preventive medicine conducted a preliminary evaluation of 10 online health-related newspaper articles with draft questions for the newly developed HIQUAL. In this process, we compared the strengths and weaknesses of the existing evaluation tools with the newly selected questions.

### Completion of the Final Tool and Evaluation of Reliability and Validity

We analyzed the interrater agreement to ensure the consistency of the evaluation tool. A total of 3 interrater agreement analyses were performed in the process of revising the HIQUAL. The first and second evaluations were performed in a pilot study using a nonfinalized version of the tool with 3 articles, while the third evaluation was performed using the final version of the tool. News articles used in the assessment were randomly selected from among the most-viewed articles of the month in the health section of a portal website, which was the most popular online search engine in Korea [30]; the final evaluation was conducted in March 2018. In the first evaluation, 6 raters (3 family medicine physicians, 2 internists, and 1 obstetrician) participated [31-33]; in the second evaluation, 14 raters (8 family medicine physicians, 1 preventive medicine physician, 4 family medicine residents, and 1 representative of a patients’ organization) participated. The second evaluation was conducted after a brief face-to-face training about the HIQUAL, and the interrater agreement was confirmed for the 2 evaluations. In the case of items with low agreement, discrepancies were identified, and the questions were revised by considering the opinions of the raters during the evaluation process. Finally, the third evaluation was conducted using the final version of the HIQUAL, and 5 raters (3 family medicine physicians, 1 preventive medicine physician, and 1 representative of a patients’ organization) who had participated in the previous evaluations reviewed 3 new articles to reach an agreement.

The interrater agreement used two methods (Fleiss κ coefficient and Gwet agreement coefficient [AC]). Fleiss κ is a method used to measure the degree of agreement between two or more raters [34,35], where higher values indicate greater agreement. Gwet AC has the advantage of being able to accurately estimate population values without responding to ambient probabilities by taking changes between raters into account [36,37] and not being vulnerable to the kappa paradox [38]. The κ statistic tends to have a low value although there is strong interrater agreement; this can lead to kappa paradox and produce a biased result [39]. Gwet AC overcomes the κ limitation since it provides a stable interrater agreement and is less affected by prevalence and marginal probability; thus, it is used as a “paradox-resistant” alternative interrater coefficient [39]. Benchmarking scales of the Fleiss scale, AC statistics of 0.40 or less indicate poor agreement, 0.40-0.75 indicates a moderate to good agreement, and 0.75 or higher indicates excellent agreement [35,37,39]. For interrater agreement, the “kappaetc” package was used with the Stata version 16 (StataCorp) statistical software program.

The validity was verified by comparing the results of 3 tools: DISCERN, QUEST, and HIQUAL. The Kendall rank correlation coefficient (τ) proposed by Kendall measures the association and strength between paired observations [40]. The Kendall τ has better statistical properties of distribution [41] compared to other rank correlations and is easy to calculate [40]. We evaluated the 16 articles that were used in the preliminary evaluation and reliability analysis by one rater, using the DISCERN, QUEST, and HIQUAL tools. We divided the articles into 3 categories: treatment-related, diagnosis-related, and prevention-related; a total of 9 correlational tests were performed. The Kendall τ correlation coefficient returns a value of 0 to 1, where 0 indicates no relationship and 1 indicates perfect relationship. As a rule of thumb, the strengths of the correlation categories are as follows: 0.00 to 0.30 (0.00 to −0.30) indicates a negligible correlation, 0.30 to 0.50 (−0.30 to −0.50) indicates a low positive (negative) correlation, 0.50 to 0.70 (−0.50 to −0.70) indicates a moderate positive (negative) correlation, 0.70 to 090 (−0.50 to −0.70) indicates a high positive (negative) correlation, and 0.90 to 1.00 (−0.90 to −1.00) indicates a very high positive (negative) correlation [42]. For the validity tests, we used Stata version 16 and SPSS version 26 (IBM Corp).

## Results

### Tool Overview

**Figure 3:**
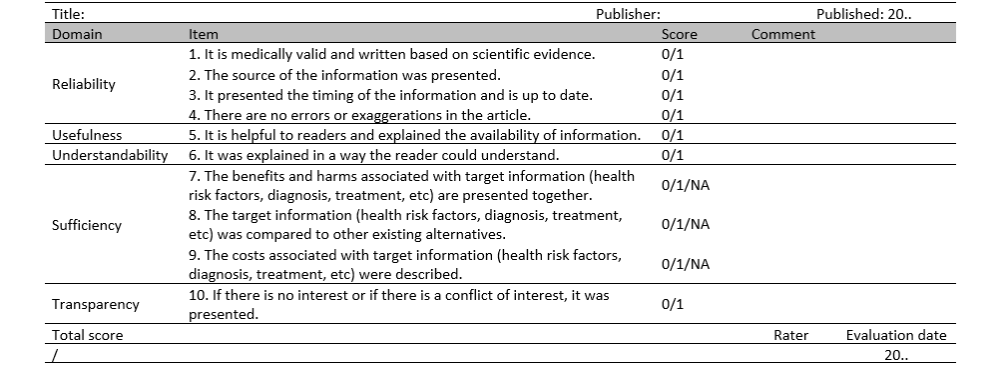
Health Information Quality Assessment Tool (HIQUAL) for health-related newspaper articles (English translated version).

The final version of the HIQUAL is presented in a table format. The newly developed tool consists of the five domains of reliability, usefulness, understandability, sufficiency, and transparency, and has 10 questions (Figure 3 and Multimedia Appendix 1). The evaluation results are divided into 3 categories—Yes (1 point), No (0 points), and Not Applicable (NA)—and the final score is calculated by adding up the corresponding scores. NA results are excluded when calculating the total score. For example, if out of the 10 questions, 7 are marked as “yes,” 2 as “no,” and 1 as “NA,” the total score is 7 out of 9 points (78%), instead of 7 out of 10 points (70%).

### Reliability Analysis

Using the HIQUAL, 5 raters evaluated 3 online newspaper articles. The interrater agreement was then analyzed (Table 1). Agreement was in the moderate to substantial range for the colorectal cancer–related article [43] (Fleiss κ=0.49, SE 0.11; Gwet AC=0.74, SE 0.13) and in the substantial range for the article introducing obesity genetic testing [44] (Fleiss κ=0.63, SE 0.28; Gwet AC=0.86, SE 0.10). In contrast, the article introducing the study of the changed hypertension diagnostic criteria showed a low level of agreement for Fleiss κ at 0.20 (SE 0.10) but a substantial agreement for Gwet AC at 0.75 (SE 0.13) [45]. For this article, the results of 4 raters’ evaluations, excluding 1 representative of a patients’ organization, showed a moderate agreement with a value of 0.40 (SE 0.19) for Fleiss κ, while showing an excellent agreement for Gwet AC at 0.85 (SE 0.11). With a reanalysis of the evaluation results, we confirmed that the level of agreement increased when we examined the results of the 4 medical specialists and excluded those of the nonmedical rater.

In terms of the statistical values of agreement, the coefficients for Fleiss κ were lower than those for Gwet AC. However, the overall percentage of agreements, including that of the third article with the lowest interrater agreement, was higher than 0.70. Fleiss κ is a model developed in Cohen κ, which may show the kappa paradox [35]. Gwet AC provides a more stable agreement than κ [39]; hence, in this case, it may be more appropriate to select the Gwet AC statistic [38,39]. For the value of Gwet AC, all 3 articles showed a high, close to excellent agreement.

**Table 1:** The interrater agreement based on 3 articles with 5 raters.

Statistic	Coefficient (SE)		
	Article 1	Article 2	Article 3
Scott/Fleiss κ	0.49 (0.11)	0.63 (0.28)	0.20 (0.10)
Gwet agreement coefficient	0.74 (0.13)	0.86 (0.10)	0.75 (0.13)
Percentage of agreement	0.79 (0.09)	0.90 (0.07)	0.78 (0.10)

### Validity Analysis

The results of the validity tests are shown in Table 2. Sixteen online newspaper articles evaluated using Kendall τ showed a moderate to high correlation between the tools. For the 16 articles as a whole, the lowest correlation was obtained when comparing HIQUAL to QUEST, with the lowest at 0.69 and the highest at 0.72; there was a strong correlation when comparing HIQUAL and DISCERN. With treatment-related articles, the comparison between HIQUAL and QUEST was the lowest at 0.59, and the comparison between QUEST and DISCERN showed the highest correlation at 0.75. The lowest correlation was between QUEST and DISCERN (0.48) for the articles with content on topics other than treatment, and the highest correlation was between HIQUAL and DISCERN (0.67).

**Table 2:** Validity test with Kendall τ, SE, 95% CI, and *P* value of each test for health-related articles.

Article category		Kendall τ (95% CI)
**Total articles (n=16)**		
	HIQUAL^a^ vs DISCERN	0.72 (0.49-0.86)
	HIQUAL vs QUEST^b^	0.69 (0.44-0.84)
	QUEST vs DISCERN	0.70 (0.45-0.84)
**Treatment articles (n=7)**		
	HIQUAL vs DISCERN	0.62 (0-0.90)
	HIQUAL vs QUEST	0.59 (0-0.89)
	QUEST vs DISCERN	0.75 (0.22-0.94)
**Diagnosis and prevention articles (n=9)**		
	HIQUAL vs DISCERN	0.67 (0.22-0.88)
	HIQUAL vs QUEST	0.65 (0.19-0.87)
	QUEST vs DISCERN	0.48 (0-0.80)

^a^HIQUAL: Health Information Quality Assessment Tool.

^b^QUEST: Quality Evaluation Scoring Tool.

## Discussion

### Principal Results

In this study, we developed a novel tool to evaluate the quality of health information in online newspaper articles by reviewing previous studies and existing tools. The HIQUAL consists of 5 domains, namely reliability, usefulness, ease of understanding, sufficiency, and transparency. We found the HIQUAL to have high interrater agreement. After evaluating a total of 16 online newspaper articles, the HIQUAL was highly correlated with two other tools—DISCERN and QUEST. The results of the analysis, divided into treatment articles and diagnosis and prevention articles, also showed similar results to the overall analysis.

### Comparison With Prior Work

In the process of developing the HIQUAL, a variety of previously developed quality assessment tools were compared. Among them was the DISCERN instrument, which consists of 16 questions and assesses the quality of treatment information for diseases; several studies have demonstrated its validity and reliability [26,46]. In Korea, Park et al used the translated version of the tool to evaluate the quality of health information websites that provide information on diseases such as breast cancer, asthma, depression, and obesity [47]. In addition, DISCERN was also used to evaluate websites that provide information on colorectal cancer [48], hepatitis B [49], and precocious puberty [50]. The DISCERN instrument has the advantage of being useful both to experts and the general public for conducting systematic comprehensive assessments, but it has not yet been validated in Korea and may be difficult to apply to information other than that relating to diseases and treatment [19,47]. The HONcode consists of 8 ethical codes to follow when providing information and has been used in Korea for evaluating online medical information on diabetes and thyroid cancer [51,52]. The code of ethics includes information delivery entities, sources of information, and justification items; however, the limitation is that these items do not guarantee the accuracy of the content. MedCERTAIN is part of an international project for the safe use of the internet, which requires health information providers to comply with its standards and assess compliance, based on items corresponding to standard metadata [53]. In Korea, it has been used to evaluate websites that provide information on dementia [54]. The HONcode and MedCERTAIN are better suited for evaluating platforms or websites that provide information, rather than evaluating individual online newspaper articles. QUEST was recently developed for evaluating online health information and has proven to be comparable to DISCERN [6]. This tool uses the 6 criteria of authorship, attribution, conflict of interest, currency, complementarity, and tone. However, the questions related to usefulness and understandability in HIQUAL’s criteria were not used in QUEST. In addition, QUEST uses indirect evaluations on the basis of the tone to assess for exaggeration or error, which may facilitate more objective evaluations by nonexpert evaluators but may also lead to a somewhat less accurate assessment.

Since the number of consumers using internet health information has increased in Korea, Son presented criteria for quality evaluation based on prior studies that reviewed domestic and foreign health information websites [11]. This study faced limitations in applying these criteria because the actual assessment was not carried out. Kim et al developed user-specific (professional, operator, and general public) assessment tools for internet health information and have confirmed the reliability of these tools with the public and experts [12]. However, it is difficult to apply the tool to other types of media because it is intended to evaluate websites only. The tool developed by the Korean Medical Association consists of 14 questions across 5 categories: whether the information was reliable (reliability), whether it was helpful to readers (usefulness), whether the readers understood the contents (understandability), whether the information was provided without omission (completeness), and whether this health information was organized for public interest (publicity). Although experts were asked to evaluate the suitability of each item, the development process of the evaluation items, validity, and reliability were not demonstrated [13]. The Korean Medical Association also evaluated the health information of newspaper articles and the internet through its own standards to identify the health-related information sought by consumers. There was a limit to the representativeness of the subjects who conducted the evaluation based on the proposed criteria.

A variety of tools have been used, but most of them are limited in the purpose and objective of evaluation and may not be suitable for evaluating online newspaper articles. Newspaper articles cover a wide range of content, ranging from diagnosing, treating, and preventing diseases to health care and new scientific discoveries. It should be considered that insufficient information, as well as information supported by scant evidence, can be communicated to an unspecified number of people in this process. Additionally, it is also necessary to convey sufficient information from an objective and independent perspective, as well as an appropriate understanding of the uncertainty of scientific research [17]. However, according to a study in the United States that analyzed online newspaper articles on drugs, many articles did not provide sufficient information, including the side effects and the cost of the medications [20]. There are nonprofit websites such as HealthNewsReview.org that have created their own criteria to address these problems, but the evaluations by this site are currently suspended. An analysis of articles on the new guidelines for diagnosing high blood pressure using those criteria, released in 2017, showed that only 33 of the 100 articles mentioned the benefits and risks that could arise from the changed guidelines, while only 2 articles mentioned conflicts of interest [55]. The validity and reliability of the criteria were not identified, but as these criteria also target online articles, they were considered in the development process of this study.

### Limitations

There are points to consider when applying the HIQUAL. First, it is aimed at health-related online newspaper articles, so it is difficult to apply it to other forms of media or information. A tool that can cover a variety of evaluation targets may degrade its own accuracy and value, and well-made existing tools such as DISCERN and HONcode also limit their evaluation targets. Consequently, a tool developed in accordance with the characteristics and purpose of the evaluation target can be determined well in advance to evaluate the evaluation medium. Furthermore, it can be used more effectively when applied to articles that require neutral and sufficient information delivery, such as new medical findings or treatments, than to articles that convey well-known universal knowledge. With the evolution of technology over time, the issues of inaccurate online information will continue to arise, so our tool can be useful in targeting online newspaper readers.

The second limitation is the problem of the users. The result of the interrater agreement of the third article, which had a lower level of agreement than the other articles, was analyzed. We confirmed that the level of agreement increased when we examined the results of 4 medical specialists. To use this tool properly, judgment on medical validity or errors is necessary, and considering this, it is desirable for medical doctors or professionals in the field of health care to participate in the evaluation. In addition, as the results of the interrater agreement show, there may be some differences in the results of the assessment among raters. Therefore, in such a case, quality evaluation may be considered by two or more evaluators individually, and a final evaluation may be derived through consultation. During the actual application, it may also be useful to train the raters in advance to fully understand the assessment tools or to organize and operate an evaluation group where raters who are familiar with the tools can continue to participate.

Third, although the criteria and tools have been revised and identified by repeating the process of verifying reliability, the fact that the confirmation of the reliability and validity of the final completed tool was made using a relatively small number of articles may constitute another limitation. The fact that the final evaluation was made by a small number of experts and that they were not a representative group could also be a limitation of this study.

### Conclusions

This study developed a new evaluation tool, the HIQUAL, for performing quality assessment of health-related online newspaper articles. For more effective use of the tool, it is desirable to establish a system that continuously monitors and evaluates health-related articles and delivers evaluation results to consumers so that they can make accurate judgments. Moreover, this tool could help information producers, such as journalists or reporters, produce quality health-related information articles. With the quality assessment tool, data producers can provide accurate and understandable information for online health-related articles. Using the HIQUAL, it will be possible to establish a platform that conducts continuous evaluations and regularly publishes the results, giving audiences access to high-quality health-related online newspaper articles. In addition, this tool will guide practitioners in the medical field in advancing sound strategies for disseminating health information among the general public and promote collaboration between experienced medical practitioners and news sites. Against the backdrop of the increasing number of health-related newspaper articles, as well as concerns about their quality and accuracy, this tool may be useful for assessing the quality of online health information in Korea.

## Appendix

Multimedia Appendix 1 Quality assessment tool for health-related newspaper article (Korean version).

## Abbreviations

AC: agreement coefficient
DQ: data quality
HIQUAL: Health Information Quality Assessment Tool
HONcode: Health on the Net Foundation Code of Conduct
QUEST: Quality Evaluation Scoring Tool
