# Supplementary material for: A Health Information Quality Assessment Tool for Korean Online Newspaper Articles: Development Study
Source: J Med Internet Res. 2021 Jul 29;23(7):e24436. doi: 10.2196/24436 (PMC8367132; doi:10.2196/24436)
Supplement: Multimedia Appendix 1 [file jmir_v23i7e24436_app1.pdf]

Additional file

Table S1. **Quality assessment tool for health-related newspaper article (Korean version)**

기사 제목:	언론사:	게재일: 20 . .

분류 (Domain)	항목 (Item)	점수 (Score)		평가 이유 (Comment)
**신뢰성 (Reliability)**	1. 의학적으로 타당하며 과학적 근거를 바탕으로 했다.	0/1		
	2. 정보의 출처를 제시했다.	0/1		
	3. 정보의 시기를 제시했으며 최신 내용이다.	0/1		
	4. 오류나 과장이 없다.	0/1		
**유용성 (Usefulness)**	5. 독자에게 도움이 되며, 정보의 활용 가능성에 대해 설명했다.	0/1		
**이해의 용의성 (Understandability)**	6. 독자가 이해할 수 있게 설명했다.	0/1		
**충분성 (Sufficiency)**	7. 대상 정보(건강위험요인, 진단, 치료 등)와 관련된 이득과 위해를 함께 제시했다.	0/1/NA		
	8. 대상 정보(건강위험요인, 진단, 치료 등)를 대체 가능한 다른 방법과 비교해 설명했다.	0/1/NA		
	9. 대상 정보(건강위험요인, 진단, 치료 등)와 관련된 비용에 대해 설명했다.	0/1/NA		
**투명성 (Transparency)**	10. 이해관계가 없거나, 이해관계 상충이 있는 경우 이를 제시했다.	0/1		

**총점(Total score)**[1)]		**평가자**	**평가일**
/			**20 . .**
